# Male Sex Bias in Immune Biomarkers for Tuberculosis

**DOI:** 10.3389/fimmu.2021.640903

**Published:** 2021-03-16

**Authors:** Graham H. Bothamley

**Affiliations:** ^1^TB Team, Department of Respiratory Medicine, Homerton University Hospital, London, United Kingdom; ^2^Faculty of Infectious and Tropical Diseases, London School of Hygiene and Tropical Medicine, London, United Kingdom; ^3^Blizard Institute, Barts and The London School of Medicine and Dentistry, Queen Mary University of London, London, United Kingdom

**Keywords:** sex-bias, tuberculosis, biomarkers, interferon-gamma release assay, delayed-type hypersensitivity, diagnosis, prognosis

## Abstract

Males have a bias toward developing sputum smear-positive pulmonary tuberculosis, whereas other forms of the disease have an equal sex ratio. Immune responses are known to be affected by estrogen and testosterone. Biomarkers may therefore be affected by these hormones, especially between 16 and 45 years of age when the differences are most marked. Using large data sets, we examined whether the male bias was significant in terms of diagnosis or predictive ability for the development of disease in those exposed to tuberculosis. Despite the large numbers, the need to specify homogeneous population groups for analysis affected the statistical power to discount a useful biomarker. In general, males showed higher interferon-gamma responses to TB antigens ESAT-6 and CFP-10, whilst females had stronger tuberculin responses in those with sputum smear- and culture-positive tuberculosis, but smaller responses in those who were screened for tuberculosis and who did not develop disease. Importantly, in contacts of sputum smear-positive pulmonary tuberculosis, more males who did not develop tuberculosis had tuberculin skin tests in the range between 10 and 14 mm, suggesting that sex-specific cut-offs might be better than general cut-off values for determining who should receive preventive treatment. Immunocytochemistry of the tuberculin responses correlated with cell numbers only in females. Total and anti-lipoarabinomannan IgM antibody levels were lower in males, whereas total and anti-BCG IgE antibody levels were higher. Evaluation of biomarkers should take account of the spectrum of tuberculosis and male sex bias for sputum smear-positive pulmonary tuberculosis. These findings improve our understanding of how immune responses contribute to the pathogenesis of infectious tuberculosis as well as suggesting clinical applications of the differences between the sexes.

## Introduction

Biomarkers are “intended as substitutes for a clinical endpoint… to predict clinical benefit (or harm) based on … scientific evidence” (1). In tuberculosis, mycobacterial culture and identification of the species is the gold standard for diagnosis. The detection of DNA (e.g., Xpert MTB/RIF) or proteins (e.g., MPT64) found only in *Mycobacterium tuberculosis* (Mtb) can be seen as part of this process. One step removed is to use the immune system to amplify the signal, by measuring immune responses from T cells (e.g., interferon-gamma release assays, IGRAs) or B cells (antibody to epitopes or antigens restricted to Mtb). The next step removed is to measure T cell or antibody responses to antigens that contain both specific and cross-reactive antigens (tuberculin purified protein derivative—PPD, Antigen60 or sonicated extracts of Mtb). A further step back may measure total antibody levels or inflammatory markers. Such proteomic measures may be involved in the causal outcome (clinical disease requiring treatment), to distinguish those forms of tuberculosis (TB) which require treatment compared to those that do not. Proteomic measures parallel clinical judgment from chest radiographs and symptoms, all of which may recommend further medical specific investigations for TB. However, at this distance from the causative organism, such markers may also represent tissue damage or be merely bystanders.

The term “subclinical disease” is variously used to identify those who have TB disease but either have no symptoms or who were only identified by active case finding. Clinicians would also use this term for those who present with negative bacteriology and a normal chest X-ray, who later develop active disease, e.g., those detected in contact tracing who then over the period of observation (usually 60–90 days after their first visit) develop disease that can be diagnosed microbiologically. This form of disease is common in those with HIV infection, where treatment with antiretroviral therapy (ART) reveals active TB. Separate to this category are those close contacts of an infectious case of TB who show immunological evidence of exposure to Mtb (loosely term latent tuberculosis infection—LTBI) and are offered preventive treatment or radiological follow-up over a year. “Incipient” tuberculosis, where there is “metabolic activity to indicate ongoing or impending progression of infection” (2), would include those with LTBI and raised inflammatory markers, including cytokines, or a signature transcriptome or metabolome. The term “diagnostic utility” includes identifying new cases of active TB for full treatment, those with recent contact and those screened for TB who are most likely to develop active for preventive treatment (better termed “prognostic utility”), and those where a combination of immunological, transcriptomic and proteomic tests suggests “incipient” TB for a clinical decision as to the mode of treatment.

The hypothesis was that immune responses known to be affected by estrogen and testosterone might affect the level and diagnostic utility of a biomarker especially in sputum smear- and culture-positive tuberculosis (S+PTB), where the male to female sex-ratio is of the order of 2:1 (3–8, and annual reports thereafter). In this analysis, the influence of sex-specific effects on T cell and antibody responses will be explored using data from publications whose purpose was to establish the role of these biomarkers in predicting or establishing a diagnosis of active TB.

## Methods

### Data Sources

IGRA data were from the UK PREDICT TB study (9,870 records, a cohort study with follow-up of 2.5–7.6 years) (3), NIHR 4147 Blood Tests in Tuberculosis III (945 records, a cohort study with follow-up of at least 8 years) (4), Epitope-Specific Antibody Levels in Tuberculosis (747 records, a cohort study with follow-up of at least two years) (5–8) and an in-depth study of Indonesians affected by tuberculosis and leprosy (349 records, a cross-sectional study) (9–11). These publications provide details on the methods of measurement of the T cell assays, immunocytochemistry and antibody levels and the ethical approvals of each study.

### Patient Selection

Children under 16 years of age were excluded from the data analysis. Where possible, females were selected as aged 16–45 years to exclude post-menopausal women without high estrogen levels. The reduction of testosterone with age is less abrupt and therefore analyses are specified as to whether the same cut-off as for females were used or whether the adult male population > 16 years was used. Pregnancy was an exclusion factor for the UK PREDICT TB study (3) and for analysis of other data.

Active tuberculosis was limited to those with sputum smear- and culture-positive pulmonary tuberculosis (S+PTB), as this form of tuberculosis is responsible for the difference in incidence between the sexes. However, one analysis looks at a combined population of smear-positive and smear-negative culture-positive pulmonary tuberculosis patients. The aim was to avoid any bias toward males, which often occurs due to the ease of sputum smear examination.

### Definition of Recent and Pre-Existing TB Exposure

Recent infection was defined as having a household contact of sputum-smear-positive pulmonary tuberculosis, with a positive IGRA, without HIV co-infection or previous tuberculosis. For more distant exposure, migrants from countries with an incidence of tuberculosis >100 per 100,000, not born in the UK, without HIV co-infection, recent contact with or previous TB were selected.

### IGRAs

In assessing the QuantiFERON Gold-in-Tube (QFT) data, negative controls were assessed if ≤ 8 IU/mL (10 IU/ml = 12.04 ng/mL), as per the manufacturer's standard operating procedure. Similarly, only mitogen positive controls were evaluated if > 0.5 IU/mL; all values in the 1000s were eliminated as being probably due to a transcribing error. Indeterminate results were not included in the denominators and did not contribute to the analysis. Cut-offs were determined by the manufacturer's cut-off (0.35 IU/mL for QFT) and the upper limit of the dilution curve for measuring IFNγ (≥ 10 IU/mL). The corresponding values for the TB-SPOT.*TB* test were defined according to the manufacturer's standard operating procedure as a negative control with ≤ 10 spots, an adequate positive (with mitogen) control as ≥ 20 spots and a positive test as ≥ 8 spots above the negative control; strong reactors were defined as those tests with > 100 spots. Borderline tests were used only in assessing the prognostic utility, but were usually excluded together with indeterminate tests from the denominators.

### Tuberculin Responses

Tuberculin responses were grouped by mm of induration as in the ATS guidelines (12). The majority of responses were to tuberculin-PPD RT23. New tuberculin was prepared as a sonicated extract of Mtb, thereby including non-secreted proteins, lipid and polysaccharide antigens compared to tuberculin-PPD (13), and was used in the Indonesian study data and in examining the immunocytochemistry of the response to Mtb antigens (11). Where the areas of induration were used, these were calculated by multiplying the measurements in two axes (without dividing by π/2, if indurations were considered perfect ellipses). The “cut-offs” for immunocytochemistry were determined in relation to the delayed hypersensitivity responses from patients and controls, being the point at which the CD4+, CD8+, and CD14+ cell numbers began to rise; these corresponded to between 8 and 9 mm of induration to new tuberculin.

### Antibody Titers

Total IgM, IgG, and IgA were measured by laser nephelometry (11) and IgE levels by radioimmunoassay (10); cut-off titers were determined from normal reference ranges. Anti-BCG IgE levels were measured by radioallergoabsorbent test after competition with purified BCG antigen (10). IgM, IgG, and IgA levels to purified antigens were measured by ELISA (6) and epitope-specific antibody levels measured by a competition assay using monoclonal antibodies to the species-restricted epitopes (5, 7–9); cut-off titers were determined as the mean + 2SD of control samples. As data were normalized by log. transformation, zero values were noted separately under “diagnostic utility” in the tables.

### Statistical Analysis

Statistical analysis was performed using 2020 GraphPad Software. Student's t-test was employed for normalized data and the chi-squared test for diagnostic utility. Where the standard deviation was large, suggesting that the data had not been sufficiently normalized by log. transformation, the Mann-Whitney *U*-test was used and *P*-values then relate to the latter test. Pearson's rank correlation was used to compared log. transformed values of PPD and new tuberculin, Spearman's rank correlation to compare antibody titers.

For diagnostic utility, a table of the number required for a power analysis relating to differences in sensitivity has been supplied (Table 1). *P*-values are given only where *P* < 0.1 (comparable to a false detection rate of < 10%). The cut-offs were defined for each test as the manufacturer's chosen endpoints, the normal ranges or from the mean + 2SD for new tests. For the diagnosis of sputum smear- and culture-positive pulmonary tuberculosis, the discrimination of active from LTBI by IGRAs and tuberculin has not been calculated, noting the poor specificities from many past studies. The prognostic utility for predicting the development of TB was compared between males and females for sensitivity and specificity of the specified criterion. Receiver-Operator Characteristic (ROC) analysis was conducted using the web-based calculator of John Hopkins University, Baltimore (http://www.jrocfit.org); differences between AUCs were assessed for significance using the online calculator http://vassarstats.net/roc_comp.html.

**Table 1:** Sample sizes to detect differences according to sensitivity at P < 0.05 and power of 80%.

	**Sensitivity of test in females**								
	**10%**	**20%**	**30%**	**40%**	**50%**	**60%**	**70%**	**80%**	**90%**
Difference^*a*^									
+5%									
Male	1004	1623	2053	2295	2348	2213	1890	1378	676
Female	502	812	1026	1148	1174	1106	945	689	338
+10%									
Male	287	431	529	579	582	538	447	308	NA
Female	144	216	264	290	291	269	224	154	
+15%									
Male	142	202	240	258	255	231	186	119	NA
Female	71	101	120	129	128	116	93	60	
+20%									
Male	87	118	137	145	141	124	96	NA	NA
Female	44	59	68	72	70	60	48		

a *Assumes enrolment at usual male to female ratio in S+PTB of 2:1. + = males > females*.

*Where male sensitivity < females, same numbers apply but using sensitivity of test in females = (100–given sensitivity) in table*.

## Results

### Developing the Hypotheses

We conducted a systematic review of sex-related immune responses, using the comprehensive MeSH terms “estrogen,” “testosterone,” “sex,” “immune response,” without time limit. Titles and abstracts underwent a first screen; relevant articles were selected for a second screen, which included full text review. Most hormone-induced sex-specific immune responses have been studied in animal models and in relation to non-infectious diseases, such as autoimmunity (rheumatoid arthritis, lupus, extrinsic allergic encephalitis/multiple sclerosis), estrogen receptor-α^+^ breast cancer or infections, such as lymphocytic choriomeningitis virus. The predictions listed in Table 2 are therefore somewhat removed from the topic of human TB. The testing of these hypotheses in sputum smear- and culture-positive pulmonary tuberculosis may indicate whether further exploration of particular immune responses, in order to understand the male predominance of smear-positive pulmonary tuberculosis, is merited.

**Table 2:** Hypotheses derived from literature review of hormone-induced sex-specific immune responses.

**Immune response**	**Diagnostic measure**	**Predictions^**a**^**
**T cell**		
Spontaneous IFNγ production	Negative control IGRA	F > M (14)
Mitogen IFNγ production	Positive control IGRA	F > M (15, 16)
Antigen-specific IFNγ production	IGRA test result	F > M (16)
Delayed hypersensitivity		
Tuberculin response	48 h induration to tuberculins	F > M
	Blood flow velocity	F > M
	Central necrosis/slow blood flow	Uncertain
Histopathology		
	CD4+ T cells	F > M (17, 18)
	CD8+ T cells	M > F (19, 20)
	Macrophages (CD14+)	M > F
**Antibody**		
Total	Globulin (g/L)	F > M (21, 22)
	IgM	F > M (22)
	IgG	No difference
	IgA	No difference
	IgE	M = F (23) or
		M > F (24)
Anti-mycobacterial antibody	IgEsp	M > F (25)
IgG antibody to purified antigens^*b*^ (21)	38 kDa	F > M (26)
	32 kDa/Antigen 85B	Or M >F (27)
	30 kDa/Antigen 85A	
	Hsp16/16 kDa/	M > F (28)
	Hsp65	
	19 kDa	M > F (28–31)
	Lipoarabinomannan (LAM)	M > F (28–31)
Epitope-specific antibody	Competition assay with mAbs	
	Protein antigens	F > M (26)
	Anti-LAM antibodies	M > F (28)

a *Hormone-induced sex-specific immune responses have been studied in models not closely related to tuberculosis*.

b *More antigens and higher antibody levels are found in patents with S+PTB, especially if there are lung cavities, both features of male disease*.

One of the difficulties in evaluating data from patients with TB is that selection of patients with different forms of the disease will affect the conclusions, depending on how many have S+PTB, where the male bias will then affect the data (32, 33). This can be especially problematic when comparing LTBI with active disease, where those with LTBI will have an equal sex ratio and active disease has a male predominance, e. g. NK cells are less in number in females than males and therefore associations with active TB may be sex-specific (34, 35).

### T Cell Responses

#### Smear- and Culture-Positive Pulmonary Tuberculosis (S+PTB)

In general, IGRAs are not recommended for patients with symptoms and investigations suggesting pulmonary tuberculosis—a sputum smear is usually obtained! For this reason, there are few data and certainly numbers are insufficient to gain enough power to avoid a type II error of attributing a non-significant value as excluding the hypothesis of a difference between the sexes, even for a 20% difference (see Table 1).

**Figure 1:**
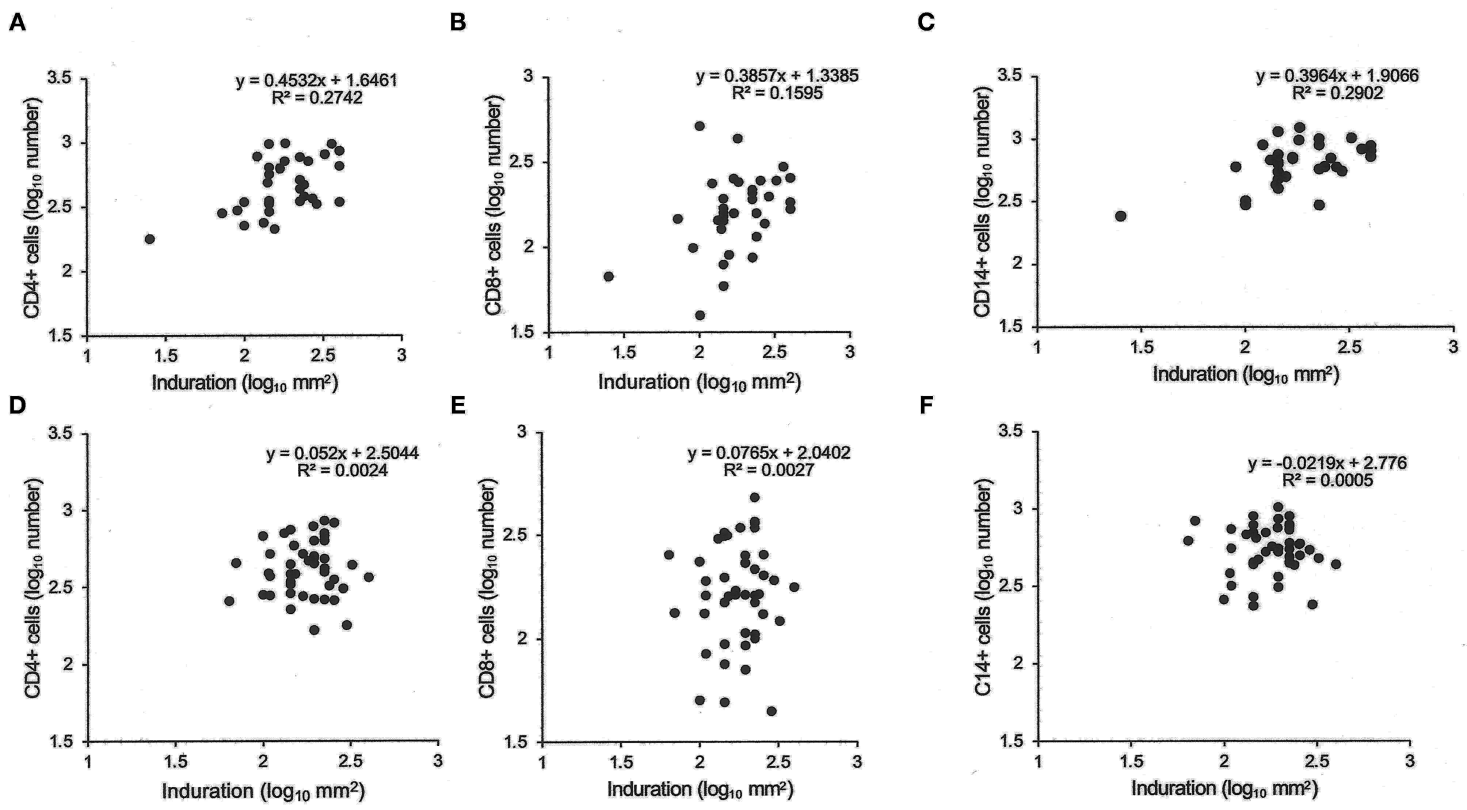
Tuberculin responses and immunocytochemistry. The cell number in biopsies of tuberculin responses were counted after labeling for CD4 **(A,D)**, CD8 **(B,E)**, and CD14 **(C,F)**. Samples from female patients are labeled **(A–C)** and male from **(D–F)**. Pearson's correlations were significant (*P* < 0.05) for female samples but non-significant for male samples.

Similarly, tuberculin testing would not normally be performed in those with sputum smear-positive pulmonary tuberculosis (S+PTB), except as part of a formal study, such as that in Indonesia comparing PPD-RT23 with new tuberculin [Supplementary Figure 1, (11)]. With both tuberculins, the diameters of induration were slightly higher in females, but only with new tuberculin did the differences approach statistical significance (Table 3: *t* = 1.8, *P* = 0.07). Females showed a greater blood flow velocity (*t* = 2.12, *P* = 0.037). Statistical significance had been observed, but only in HLA-DR15-negative subjects in the Indonesian study (tuberculin, females median 16.75 (range 4 to 22) vs. males 15 (11 to 20), Mann-Whitney *U*-test, *P* = 0.016; blood flow velocity, females 7.3 ± 2.3 vs. males 5.7 ± 1.8, *t* = 2.12, *df* 31, *P* = 0.04) (36). The immunocytochemistry data showed a correlation between induration and CD4+, CD8+, and CD14+ cell numbers in females but not in males (Figure 1).

**Table 3:** Sex and T cell responses in sputum smear- and culture-positive pulmonary tuberculosis (and sputum smear-positive OR negative but culture-positive pulmonary tuberculosis only where indicated).

**Test**	**Females**		**Males**			**“Diagnostic” utility n (%)**			
	***n***	**Values**	***n***	**Values**	***P*-value**	**Criterion**	**Females**	**Males**	***P*-value**
**IGRA-QFT**^*a*^									
TB antigen (positives only: log ± SD)	16		37			0-0.34 IU/mL	1 (6.3)	3 (8.1)	NS
	16	0.62 ± 0.549	34	0.54 ± 0.49	NS	≥ 0.35 IU/mL	15 (93.8)	34 (91.9)	NS
						≥ 10 IU/ml	6 (37.5)	9 (26.5)	NS
Culture-positive PTB: TB antigen (positives only; log ± SD)	32		62			0-0.34 IU/mL	5 (15.6)	6 (9.7)	NS
	27	0.55 ± 0.55	56	0.60 ± 0.57	NS	≥ 0.35 IU/mL	27 (84.4)	56 (90.3)	NS
						≥ 10 IU/ml	10 (31.3)	16 (25.8)	NS
**Delayed hypersensitivity**									
PPD-RT23 (mm induration ± SD)	57	17.4 ± 5.9	64	16.6 ± 4.9	NS	≥ 15	50 (87.7)	54 (84.4)	NS
						≥ 10	54 (94.7)	59 (92.2)	NS
						0	1 (1.8)	1 (1.6)	NS
New tuberculin (mm induration ± SD)	96	13.9 ± 3.6	115	12.9 ± 3.9	0.07	≥ 15	46 (47.9)	42 (36.5)	NS
						≥ 10	87 (90.6)	100 (87.0)	NS
						0	2 (2.1)	4 (3.5)	NS
Blood Flow velocity (V ± SD)	46	6.70 ± 2.30	55	5.81 ± 1.90	0.037				
Central slowing	46	NA	55	NA		Present	12 (26.1)	7 (12.7)	0.09
**Immunocytochemistry (new tuberculin)**									
CD4+ cells (log ± SD)	36	2.64 ± 0.23	45	2.62 ± 0.18	NS	2.82	10 (27.8)	9 (20.0)	0.09
CD8+ cells (log ± SD)	37	2.09 ± 0.39	46	2.00 ± 0.39	NS	2.22	17 (46.0)	13 (28.9)	0.11
CD14+ macrophages (log ± SD)	36	2.78 ± 0.18	45	2.73 ± 0.16	NS	3.02	2 (5.6)	0 (0)	NA

a *Includes only those aged 16-45 year (for UK PREDICT TB study diagnosis of culture-positive pulmonary tuberculosis within 2 months of first screening)*.

#### Exposure to Tuberculosis

There were few differences between males and females in their QFT results (Table 4). In migrants who did not develop tuberculosis, males showed greater spontaneous IFNγ production (*t* = 3.2, *P* = 0.0013; Table 4). Using a cut-off of ≥ 0.35/mL, more males than females also had higher values (χ^2^ = 11.3, *P* = 0.008). However, if they went on to develop tuberculosis later, no difference was detected. IFNγ values in response to mitogen were also greater in males than females. Males had a greater response to the RD1 antigens if they had a positive test and did not develop TB, whereas levels were lower, although not significantly so, in males compared to females who developed TB later. With the T-SPOT.*TB* test, the differences in titers were not significant, except for spontaneous IFNγ production in migrants who had a negative result, where again males had higher values (Table 4). Fewer male contacts of infectious tuberculosis showed a complete lack of response to tuberculin and more males had positive responses as defined by the different cut-off indurations (Table 5). Males were also more likely to have to have a tuberculin response ≥ 5 mm and less likely to be anergic (Table 5), even though males were less likely to have received BCG vaccination (χ^2^ = 10.5, *P* = 0.001). There was no effect of IGRA status on these differences between males and females.

**Table 4:** Sex and IGRAs after exposure to tuberculosis.

**Test**	**Female 16-44 years**		**Males 16-44 years**			**“Diagnostic” utility** ***n*** **(%)**			
	***n***	**Values**	***n***	**Values**	***P*-value**	**Criterion**	**Females**	**Males**	***P*-value**
**IGRA-QFT**^*a*^									
**Contacts S+PTB (number)**	297		161						
TB antigen (positive tests only—no TB; log ± SD)	51	0.48 ± 0.48	49	0.49 ± 049	NS	≥0.35	25 (16.1)^*b*^	33 (22.9)^*b*^	NS
TB antigen (positive tests only—TB; median, range)	5	2.72 (0, 9.2)	5	4.01 (1.74, 10)	NS	IU/mL	5 (71.4)	5 (83.3)	NS
**Migrants**									
Spontaneous IFNγ (negative tests only; log ± SD)	953	−0.85 ± 0.33	1051	−0.80 ± 0.36	<0.0013		95 (10.0)	145 (13.8)	0.008
Spontaneous IFNγ (positive tests only; log ± SD)	236	−0.74 ± 0.41	288	−0.66 ± 0.45	0.03	≥ 0.35/mL	44 (18.7)	63 (21.9)	NS
Mitogen IFNγ (negative tests only; log ± SD)	823	1.14 ± 0.33	941	1.21 ± 0.30	<0.0001		652 (79.2)	822 (87.4)	<0.0001
Mitogen IFNγ (positive tests only; log ± SD)	194	1.12 ± 0.33	244	1.19 ± 0.21	0.005	>10 IU/mL	130 (67.0)	185 (75.8)	0.042
TB antigen (positive tests only—no TB; log ± SD)	231	0.24 ± 0.47	287	0.39 ± 0.49	0.005		231 (20.2)^*b*^	287 (21.9)^*b*^	NS
TB antigen (TB developed: median, range)	10	2.88 (0, 8.76)	21	0.41 (0, 10)	NS	≥0.35 IU/mL	7 (70.0)	11 (52.4)	NS
**T-SPOT**.***TB***^*c*^									
**Contacts S+PTB (number)**	164		144			Positives	20 (13.0)	35 (24.8)	0.01
TB antigen [positive tests, no TB; median (range)]	20	28.5 (8, 320)	35	26 (8,195)	NS	TB later and	0	0	0
TB antigen [TB, positive tests: median (range)]	7	15 (0, 162)	5	75 (2, 164)	NS	positive	3 (42.9)	4 (80.0)	NS
**Migrants**									
Spontaneous IFNγ (negative tests; median, range)	973	0 (0, 8)	968	0 (0, 8)	0.02	Positives	0	0	NA
Spontaneous IFNγ (positive tests; median, range)	174	0 (0, 7)	268	0 (0, 8)	NS	Positives	0	0	NA
Mitogen IFNγ (negative tests; median, range)	695	50 (20, 255)	760	50 (20, 265)	NS	≥100 spots	145 (20.9)	146 (19.2)	NS
Mitogen IFNγ (positive tests; median, range)	116	50 (22, 231)	199	50 (24, 233)	NS	≥100 spots	17 (14.7)	22 (11.1)	NS
TB antigen (positive tests, no TB; median, range)	169	31 (8, 250)	263	32 (8, 398)	NS	Positives	169 (14.8)	263 (21.3)	0.00004
TB antigen (TB developed; median, range)	8	56 (16, 358)	14	25 (9, 193)	NS	TB later and positive	8 (80.0)	14 (70.0)	NS

a *Contacts of S+PTB showed no difference in negative and positive controls, nor TB antigen values*.

b *QFT denominators UK PREDICT TB study only: for contacts of S+PTB were 164 (females) and 144 (males) and for migrants TB antigen QFT were 1,144 (female) and 1,311 (male)*.

c *T-SPOT denominators UK PREDICT TB study only, excluding borderline and indeterminate results: for contacts of S+PTB were 154 (female) and 141 (male) and for migrants, spontaneous interferon-γ was higher in males (χ^2^ = 5.4 for those with visible spots compared to those with no spots, P = 0.02) TB antigen data were 1,153 (females) and 1,254 (males)*.

**Table 5:** Delayed hypersensitivity responses to tuberculin (PPD) during screening for tuberculosis.

**Test**	**Females, 16-45 years**		**Males, 16-45 years**		***P*-value**	**“Diagnostic” utility: n (%)**			
	***n***	**Induration (log mean ± SD)**	***n***	**Induration (log mean ± SD)**		**Criterion**	**Female**	**Male**	***P*-value**
**Contacts S+PTB**
No TB	207		210			≥ 15	86 (41.5)	89 (42.4)	NS
	126	1.18 ± 0.22	147	1.18 ± 0.23	NS	≥ 10	105 (50.7)	127 (60.5)	0.045
						≥ 5	123 (59.4)	142 (67.6)	0.082
						0	81 (39.1)	63 (30.0)	0.05
TB developed later	8		6			≥ 15	4 (50.0)	4 (66.7)	NS
	7	1.17 ± 0.25	6	1.26 ± 0.19	NS	≥ 10	5 (62.5)	5 (83.3)	NS
						≥ 5	7 (87.5)	6 (100)	NS
						0	1 (12.5)	0 (0)	NS
**Migrants**
No TB	1102		1183			≥ 15	157 (14.3)	177 (15.0)	NS
	514	0.92 ± 0.31	602	0.97 ± 0.27	NS	≥ 10	267 (24.2)	319 (27.0)	NS
						≥ 5	422 (38.3)	535 (45.2)	0.0008
						0	588 (53.4)	581 (49.1)	0.04
TB developed later	9		20			≥ 15	5 (55.6)	11 (55.5)	NS
	8	1.21 ± 0.13	15	1.18 ± 0.18	NS	≥ 10	7 (77.8)	13 (65.5)	NS
						≥ 5	8 (88.9)	15 (75.0)	NS
						0	1 (11.1)	5 (25.0)	NS
Treated TB	9		9			≥ 15	2 (22.2)	2 (22.2)	NS
	6	1.05 ± 0.26	8	1.01 ± 0.24	NS	≥ 10	5 (55.6)	6 (66.7)	NS
						≥ 5	6 (66.7)	7 (77.8)	NS
						0	3 (33.3)	1 (11.1)	NS

### Antibody Levels

**Figure 2:**
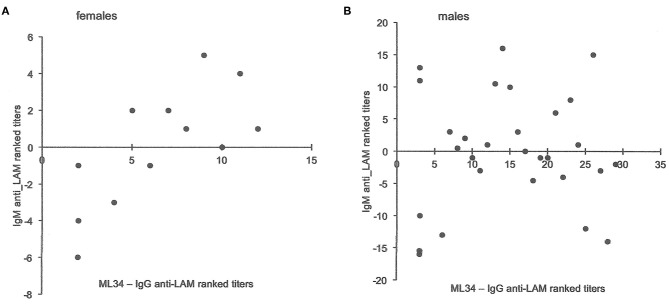
Comparison of antibody titers to lipoarabinomannan in females and males. The y-axis indicates the IgM anti-LAM ranked titers including data from Jackett et al. (6) and unpublished data from Bothamley et al. (9). For both males and females, ML34 titers had significant correlations with IgG anti-LAM titers. The x-axis is the rank for antibody to the ML34 epitope (affects binding to the two main epitopes of LAM) after subtraction of the ranked IgG anti-LAM titers. For females **(A)**, the relationship remains significant (Spearman's rank correlation: y = −0.6596x – 4.2872, ρ = 0.74, *P* = 0.01), but does not hold for males (**B**: y = −0.0696x – 0.9282, ρ = 0.06, *P* = 0.96).

Total globulin levels did not differ between males and females aged 16–45 years. Total IgM was lower and IgE higher in males with sputum smear-and culture-positive tuberculosis. IgE anti-BCG antibody levels were measured using a radioallergoabsorbent assay (RAST), measuring the inhibition of binding of specific ^125^I-labeled anti-IgE by a standard preparation of sonicated BCG-Glaxo with five dilutions tested against a standard serum to establish a standard curve (10), and showed no significant difference in titers, although titers greater than 10^3^ kU/L were more frequent in males than females (Table 6). There were limited data on IgM to purified antigens, but titers to lipoarabinomannan (LAM) were lower in males than females (*t* = 2.17, *P* = 0.048). Although IgG antibody levels to purified antigens, both proteins and LAM, and epitope-specific antibody levels to Mtb-restricted epitopes of these antigens (which do not distinguish class of antibody) showed a 1000-fold variation among individuals, no significant differences were found between the sexes. In an attempt to investigate the difference between males and females in their response to LAM, IgM, and IgG titers were ranked and compared to the ranked antibody titers to the ML34 epitope (all antibody classes assayed). The ranked IgM titers compared to ranked ML34 minus ranked IgG correlated well in females but showed no relationship in males (females, ρ = 0.74, *P* = 0.01, males ρ = 0.06, *P* = 0.96; Figure 2).

**Table 6:** Sex differences in antibody levels in S+PTB.

**Test**	**Female**		**Male**		***P*-value**	**Criterion**	**Diagnostic utility:** ***n*** **(%)**		
	***n***	**Mean ± SD**	***n***	**Mean ± SD**			**F**	**M**	***P*-value**
**Total globulin**^***a***^									
Globulin (g/L)	76	42.0 ± 7.09	167	43.6 ± 7.04	NS	> 32 g/L	71 (93.4)	120 (95.2)	NS
**Immunoglobulin class**									
IgM (g/L: mean ± SD)	49	2.30 ± 0.69	61	1.80 ± 0.74	0.0004	> 2.5 g/L	15 (30.6)	8 (13.1)	0.025
IgG (g/L: mean ± SD)	49	25.20 ± 7.80	61	24.10 ± 8.90	NS	>16 g/L	45 (91.8)	55 (90.2)	NS
IgA (g/L: mean ± SD)	49	5.36 ± 2.34	61	5.08 ± 1.92	NS	> 3g/L	40 (81.6)	55 (90.2)	NS
IgE (log IU/ml: mean ± SD)	48	2.82 ± 0.59	61	3.07 ± 0.64	0.038	> 320 U/L	33 (68.8)	50 (82.0)	NS
**IgE anti-BCG antibody RAST** (log. mean ± SD)	49	3.27 ± 0.95	61	3.52 ± 0.71	NS	>3	25 (51.0)	47 (77.0)	0.004
**IgM to lipoarabinomannan**^*a*^	11		15			0	6 (54.5)	4 (26.7)	NS
	5	2.28 ± 0.39	11	1.62 ± 0.62	0.048	>1.68	5 (45.5)	4 (26.7)	NS^*b*^

a *Only ages 16-45 years*.

b *χ^2^ = 5.7, P = 0.017 for those with any measurable titers*.

### Diagnostic and Prognostic Utility

**Figure 3:**
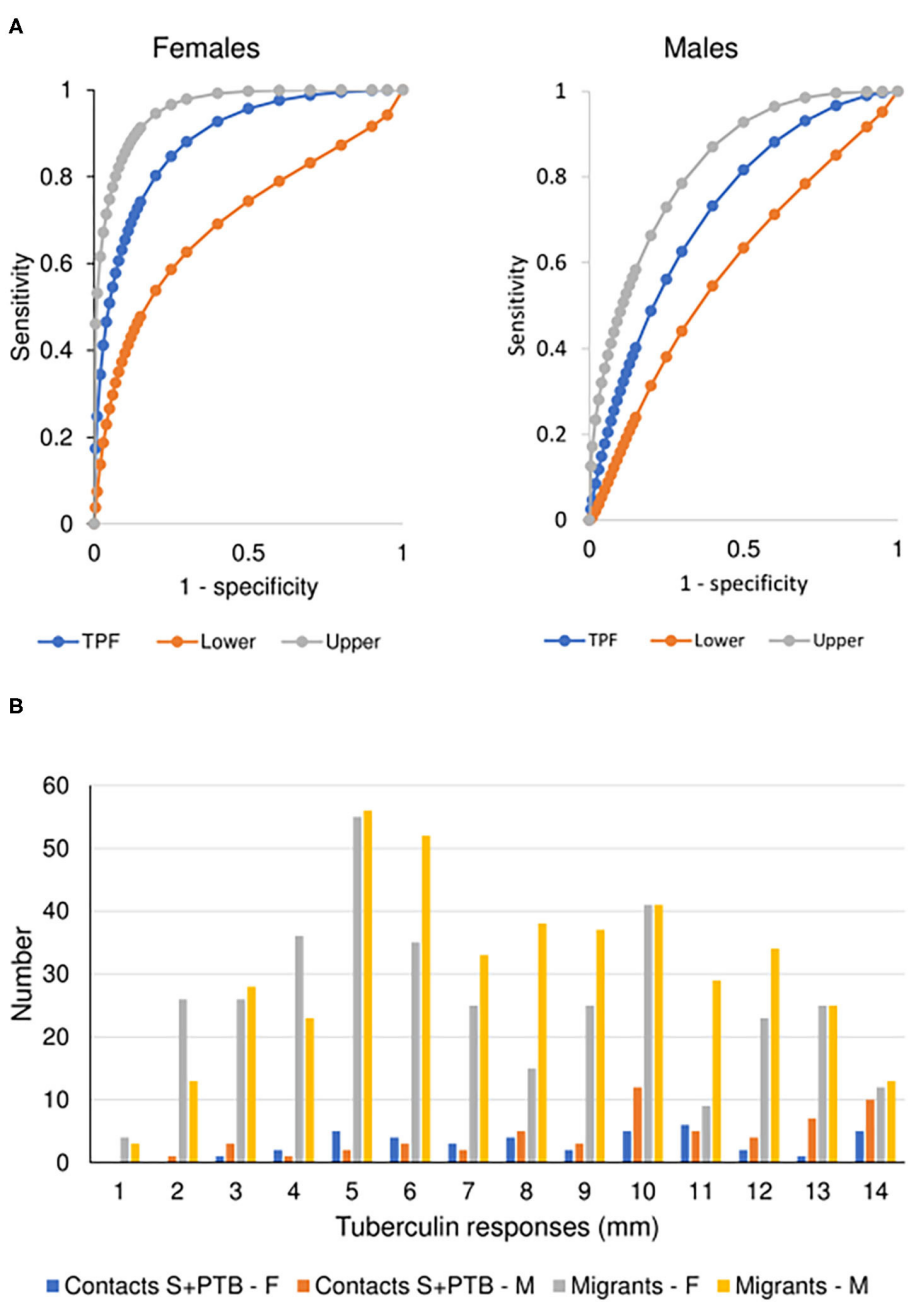
Prognostic utility of immunological tests for tuberculosis. **(A)** Comparison of ROC curves for T-SPOT.*TB* tests in migrant females and males. TPF = true positive fraction (sensitivity). The upper and lower 95% confidence intervals are given, showing overlap between the sexes. AUC females = 0.884 [10 TB cases, 1,165 no TB; AUC males = 0.727 (20 TB cases, 1,285 no TB); z = 1.65, *P* (one-tailed) = 0.49]. See Table 4. **(B)** Sex-specific differences in borderline (1–14 mm) tuberculin skin tests in subjects who did not develop tuberculosis. The majority of responses were 0 mm (contacts, female *n* = 81, male = 63; migrants, female = 588, male = 581) or ≥ 15 mm (contacts, female *n* = 86, male = 89; migrants, female = 157, male = 177). Females predominate in smaller responses (< 5 mm) and males in larger responses (10–14 mm). See Table 5.

There was no difference between males and females aged 16-45 years in the value of the tests examined in supporting the diagnosis of tuberculosis. However, male migrants were more likely to have a T-SPOT.*TB* test that recommended preventive treatment but, despite the lack of preventive treatment as specified in the protocol of the UK PREDICT TB study, were less likely to develop active disease (Figure 3A). In the UK PREDICT TB series, a cut-off of >15 spots in females would not affect the number of TB cases identified but would prevent 32 from receiving unnecessary preventive treatment. For males, increasing the cut-off to 20 would have doubled the number of missed cases of TB from 6 to 12 at a benefit of reducing unnecessary preventive treatment in 99 migrants.

For contacts of sputum smear-positive pulmonary TB who did not develop TB, males were more likely to have indurations between 10 and 14 mm than females (χ^2^ = 4.8, *P* = 0.03; Figure 3B). For male contacts of S+PTB, raising the cut-off for preventive treatment to ≥ 15 mm would prevent 38 unnecessary treatments of LTBI, without affecting appropriate preventive treatment for those who went on to develop TB. Lowering the cut-off in females to <10 mm, would add 19 preventive treatments for those who didn't develop TB but identify a further case of TB [1/9 (11%) total TB cases] for whom preventive treatment would have been valuable. In male migrants, raising the cut-off to ≥ 15 mm would prevent unnecessary chemoprophylaxis for 142, but miss one case of TB [1/20 (5%)]. For females, lowering the cut-off to <10 mm would add 110 unnecessary treatments, but identify one [1/9 (11%) total cases of TB] for whom preventive treatment would be valuable.

## Discussion

### Key Findings

Significant differences in levels of a biomarker may not translate into significant differences in diagnostic or prognostic utility and *vice versa*. This was especially important when assessing zero values or non-responders, when log. transformation is required to normalize a population result of responders (see Table 4). Secondly, despite having studies with almost 10,000 participants, the requirement to test hypotheses in homogeneous populations led to numbers that were only occasionally sufficient to have enough power to draw a statistical conclusion. This was especially important in addressing questions such as the predictive power of a biomarker to establish which of the infected population might develop active TB.

In terms of immune responses, the predictions that females would exhibit a more robust T cell and antibody response to infection (28) were only partially sustained. In order to avoid the effect of males having a higher incidence of TB, a greater TB burden and more lung inflammation, only males and females with sputum smear- and culture-positive pulmonary TB (S+PTB) or with culture-positive TB irrespective of smear status within the spectrum of active disease were each compared (Table 3). In S+PTB, the tuberculin responses of males showed lower blood flow velocities and there was a tendency to smaller areas of induration compared to females. In S+PTB, immunocytochemistry showed that females, but not males, gave a positive correlation between induration and cell type. In contrast, looking at HIV-negative migrants not born in the UK without known contact with TB who did not develop active TB, males screened for tuberculosis showed fewer anergic responses and more tuberculin responses between 5 and 10 mm induration whilst females had more responses between 10 and 15 mm induration without developing TB. Males had higher IFNγ levels both spontaneously and after mitogen stimulation (QFT only) and to TB antigens (T-SPOT.*TB* only) compared to females. Males with S+PTB had higher globulin levels, but lower IgM antibody and higher IgE and anti-BCG IgE. No differences in antibody levels to species-restricted epitopes or their purified antigens were found, except for IgM to lipoarabinomannan, compared to females.

### Limitations

We have not included children on the grounds that the circulating hormonal differences between the sexes would be absent. We have not included data from those with HIV co-infection, on the grounds that the immune responses might differ due to their immune status rather than any sex-specific effect. The choice of 16–45 years was an estimation in the absence of data regarding female participants' menopause. The upper limit of > 100 spots in the T-SPOT.*TB* assay was only available for a selection of the population, as one laboratory in the UK PREDICT TB study did not measure the high control if samples had a count of > 20 spots.

A positive sputum smear usually short circuits the diagnostic process and IGRAs may therefore have indicated those with atypical features or from whom a sputum sample was difficult to obtain, but the sex ratio of tests did not differ from that of disease and there was a determined attempt to obtain immunological markers in all studies.

The selection of two homogenous populations screened for TB (household contacts of S+PTB without HIV co-infection or previous tuberculosis and migrants not born in the UK without HIV co-infection or previous tuberculosis and with no recent TB contact) for analysis showed that even in aggregated large studies, the power to detect a significant difference and exclude a type II error may still be low.

### Sex-Related Differences in Tuberculosis Incidence and Infection Rate

One of the drivers of this analysis was the fact that sputum smear- and culture-positive tuberculosis (S+PTB) is found in males more than females (37). Some have ascribed the difference to an excess of social risk factors for developing TB (38). Others have estimated social contact to suggest that males have a greater chance of being infected with TB (39). However, these analyses do not account for the form of tuberculosis. The UK national surveys of tuberculosis (40–45) indicated that only in S+PTB is there a male predominance, but no sex predominance was noted for extra-pulmonary and smear-negative pulmonary TB. These surveys have the advantage that access to healthcare is free and possible gender bias in its uptake in fact shows a female preference. A comparison between active and passive case-finding in India showed the same male predominance in those with S+PTB, again suggesting that this is a real biological difference rather than being related to healthcare access (46). Our data show that although more male migrants were identified than females, the rate of positive QFTs did not differ, although for the T-SPOT.*TB* there were more positive tests. The rate of progression to active disease in the UK PREDICT TB data (3) did not differ between the sexes for positives with either IGRA (Tables 4, 5). However, one could argue that the numbers developing TB were too low to be confident of identifying any differences between the sexes.

### Cell-Mediated Immunity

The literature had suggested that females would have greater cell-mediated immunity (47). That this generalization is not universal is exemplified by the differences between the sexes in terms of vaccine responses, where females in general exhibit better responses but there are some vaccines, such as pneumococcal polysaccharide, where males appear to have higher antibody levels and benefit more in terms of prevention of disease (21, 48). Female neonates benefited more from BCG-enhanced trained immunity in Guinea-Bissau for protection against other respiratory infections (49) and, in adults, BCG has been used to reduce autoimmunity pathology (50). With BCG vaccination, males showed a stronger cytokine response to re-vaccination but reduced systemic inflammation (51). Our data show that males with active tuberculosis had fewer IFNγ responses > 10 IU/ml if they had sputum smear- and culture-positive pulmonary tuberculosis, but including both smear-negative and smear-positive patients into a group of culture-positive pulmonary tuberculosis resulted in a non-significant difference.

The T-SPOT.*TB* test has been evaluated in healthcare workers and shown a higher percentage of positive results in males (4.26%) than females (3.12%), but the age structure and homogeneity of the populations combined could not be assessed (52). Male migrants to the United States showed higher QFT and tuberculin responses than females (53). In our studies, male migrants with a positive IGRA who did not develop TB produced more background IFNγ, more IFNγ in response to mitogens and higher IFNγ levels to the ESAT-6 and CFP-10 antigens. This suggests that males with distant sensitization to RD1 antigens who are protected against developing TB show a good IFNγ response. The differences between the two IGRAs requires explanation. The QFT does not account for cell number as the substrate for the test is whole blood. The speculation is that males have more peripheral blood mononuclear cells/mL blood capable of secreting IFNγ than females. Where the number of PBMCs is standardized, as in the T-SPOT.*TB* test, this difference is no longer apparent. On the other hand, where the number of PBMCs is standardized, either there are more antigen-specific cells that can secrete IFNγ in males, or the stimulated cells produce more IFNγ/cell in males than females.

Early findings showed that DTH responses could be suppressed in female mice and in male mice with reduced testosterone by diethylstilbestrol, a synthetic estrogen (54). Estrogen also downregulates macrophage migration inhibitory factor (MIF) (55), a pivotal cytokine in the tuberculin response (56). Testosterone increases monocyte chemoattractant protein-1 but had no effect on MIF in a randomized treatment trial of testosterone vs. strength training in men over 62 years (57). Tuberculin responses did not differ significantly between males and females, except in migrants who did not develop TB and had larger responses. The immunocytochemistry data did not show the predicted increase in macrophages in males. In S+PTB, females but no males showed a correlation between the area of induration and cell phenotypes. Detailed phenotyping of DTH responses, especially of M1 and M2 subtypes of macrophages (58–60) and gene expression with spatial information (61), could give an indication as to this unexpected difference between males and females with S+PTB in their tuberculin responses and perhaps give an insight as to why the sex ratio in S+PTB is skewed toward males.

### Humoral Responses

Hypergammaglobulinemia is a feature of TB (62). In chronic infections, low levels of IgM antibody may indicate malnutrition as much as a defect in natural antibody-producing plasma cells (63) and rare genetic defects linked to the X-chromosome where the CD40L resides and to autosomal defects (64, 65). Usually, males have an increased expression of toll-like receptor (TLR)-2 and 4 (28). The expectation would then be that antigens such as LAM would give rise to T-independent antibody more readily in males than females and class-switching might be more effective (66). IgM antibody to LAM was lower in males but IgG antibody did not differ between the sexes in those with S+PTB. IgM may also be found in immune complexes (67, 68), which appear to have a role in pathogenesis (69). Such immune complexes to LAM and other antigens in sputum smear- and culture-positive pulmonary tuberculosis might reduce circulating serum IgM antibody levels.

Anti-BCG IgG, but not IgM, levels were found to be high in patients with pulmonary tuberculosis (70). Total IgE antibody has been found to be high in TB patients, to show a negative correlation with tuberculin responses and to resolve with successful treatment (71). In our data, IgE anti-BCG levels were found to be higher in males with sputum smear- and culture-positive tuberculosis. This might indicate a greater Th2 response in males compared to females in this form of TB. Early studies had shown that protection against tuberculosis could be transferred by cells but not by serum (72). Furthermore, as the bacterial load increased tuberculin responses were increasingly anergic and antibody levels increased (73). The resistance of many Mtb antigens to degradation by professional phagocytes and the importance of non-replicating tubercle bacilli promotes a Th2 response (74). The Th2 response can be seen as part of a greater “type 2” response encompassing a range of cells in addition to T cells, many different cytokines, different macrophage and NK cell sub-types and having a basis in metabolic changes related to the degree of inflammation (75). The fact that in the same part of the tuberculosis disease spectrum differences remain between males and females, suggests that the events leading to less IgM and more IgE-specific responses occur during early immune activation. Such a traction of the immune response after tuberculosis infection toward one which is ineffective might be responsible for male preponderance of sputum smear- and culture-positive pulmonary tuberculosis. Migrants who did not develop TB showed higher IFNγ responses than females, suggesting that the problem of a Th2 immune response occurs after the disease has elicited an immune response and that a better Th1 response in the initial stages of infection in males is required to prevent progression to active disease.

### Diagnostic Utility

Although there were significant differences in levels of IFNγ between males and females, these did not affect the numbers that would have been given preventive treatment for TB. The data were insufficient to recommend any change in the definition of a positive T-SPOT.*TB* test as a prognostic agent to identify those likely to develop TB. However, the tuberculin responses did differ such that a cut-off induration of 10 mm might be desirable for females compared to a cut-off induration of 15 mm in those aged between 16 and 45 years.

## Areas for Future Study

The first is general, regarding the use of biomarkers. Many studies use a broad-brush classifying TB as a single entity for comparison with LTBI, for instance. The differences between S+PTB and smear-negative culture-positive TB or extra-pulmonary TB in terms of antibody titers and specificities has been reported before (6, 76, 77) and is noted in terms of QFT responses between sputum smear- and culture-positive pulmonary tuberculosis and sputum smear-positive or negative culture-positive pulmonary tuberculosis (Table 3). Furthermore, the inclusion of mixtures of TB patients with variable proportions of patients with S+PTB, a part of the TB spectrum that has a male predominance, may confuse sex-related differences in biomarkers with that for TB itself. Re-analysis of these data sets by site of TB disease and by sex may provide useful insights as to the validity of proposed biomarkers and the pathogenesis of infectious forms of TB.

Our data suggests that males, rather than females, appear to be better able to produce IFNγ, and stronger delayed-type hypersensitivity (DTH) except in S+PTB. Whether this unexpected reversal of expected Th1 responses is an effect of BCG vaccination should be examined in studies specifically designed to address this hypothesis.

The role of natural antibodies and B cell subsets (78) in tuberculosis infection outcomes is of interest, especially in relation to anti-LAM IgM and IgG antibodies (79, 80).

Before considering a sex-specific cut-off value for tuberculin testing or the T-SPOT.*TB* test, a much larger number of patients who develop TB is needed in order to determine whether the benefits would outweigh the risks of a delayed diagnosis of TB.

## Conclusions

This analysis suggests that the differences in immune responses between the sexes do not affect diagnostic utility. However, in deciding who should have preventive treatment for TB, males screened as contacts of sputum smear-positive tuberculosis and migrants screened for LTBI should perhaps have a higher cut-off for the tuberculin skin test. Immunologically, the difference between migrants with evidence of exposure to tuberculosis compared to the population with sputum smear- and culture-positive pulmonary tuberculosis suggests that the male predominance in the latter might be due to immune dysregulation, with poorer IFNγ responses in those who go on to develop active disease. The lack of association between induration and CD4+, CD8+, and CD14+ cell numbers in the tuberculin DTH response in males with S+PTB requires further definition. The lower levels of IgM antibody and IgM anti-LAM antibody require further exploration to define whether this is an association or causative in the poorer T cell responses in males with S+PTB.

## Data Availability Statement

The original contributions presented in the study are included in the article/supplementary material, further inquiries can be directed to the corresponding author/s.

## Ethics Statement

The studies involving human participants were reviewed and approved by the UK PREDICT TB procedures and protocol were approved by the Brent NHS Research Ethics Committee (10/H7017/14). Blood Tests in Tuberculosis III was approved by the East London and City Health Authority Research Ethics Committee (P/03/285). The Indonesian study obtained ethical approval from the ethics committees of Airlangga University, Surabaya and Dundee Medical School. Epitope-Specific Antibody Levels in Tuberculosis was approved by the Brompton Hospitals Ethics Committee (London Chest Hospital, 1985). The patients/participants provided their written informed consent to participate in this study.

## Author Contributions

The author conceived the study, performed the systematic review, the statistical analysis and drafted the manuscript.

## Conflict of Interest

The author declares that the research was conducted in the absence of any commercial or financial relationships that could be construed as a potential conflict of interest.
