# Supplementary material for: Male Sex Bias in Immune Biomarkers for Tuberculosis
Source: Front Immunol. 2021 Mar 16;12:640903. doi: 10.3389/fimmu.2021.640903 (PMC8007857; doi:10.3389/fimmu.2021.640903)
Supplement: Supplementary file 1 [file Data_Sheet_1.docx]

Supplementary Material

# Supplementary Data

Excel files:

1. Data used in producing the Tables

2. Complete data for the Indonesian data set

**
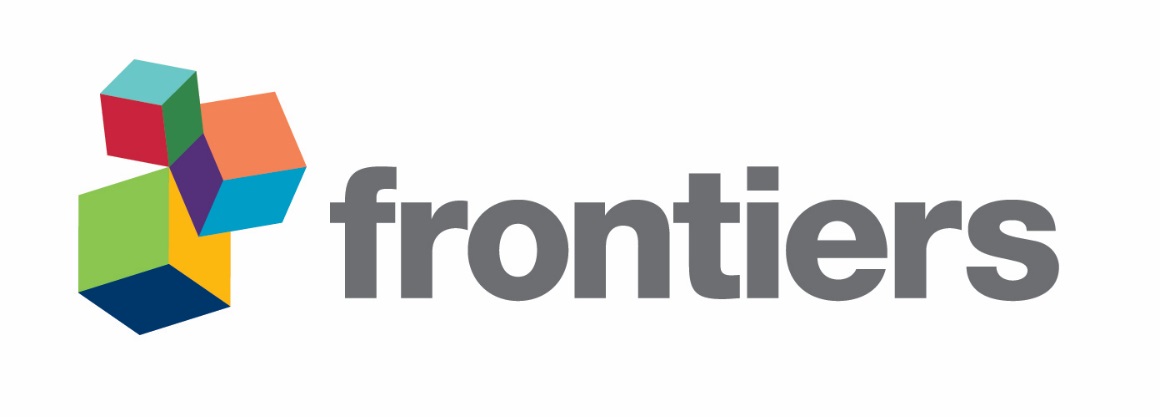
**

**Supplementary Figure 1.** **Comparison of PPD-RT23 and New Tuberculin**

Areas of induration were calculated from measurements along and across the tuberculin response, measured with the ball-point method, by simple multiplication (numbers were not divided by π/2 as would be required for a perfect ellipse).
