# Supplementary figures and images for: Male Sex Bias in Immune Biomarkers for Tuberculosis
Source: Front Immunol. 2021 Mar 16;12:640903. doi: 10.3389/fimmu.2021.640903 (PMC8007857; doi:10.3389/fimmu.2021.640903)

## Slide 1
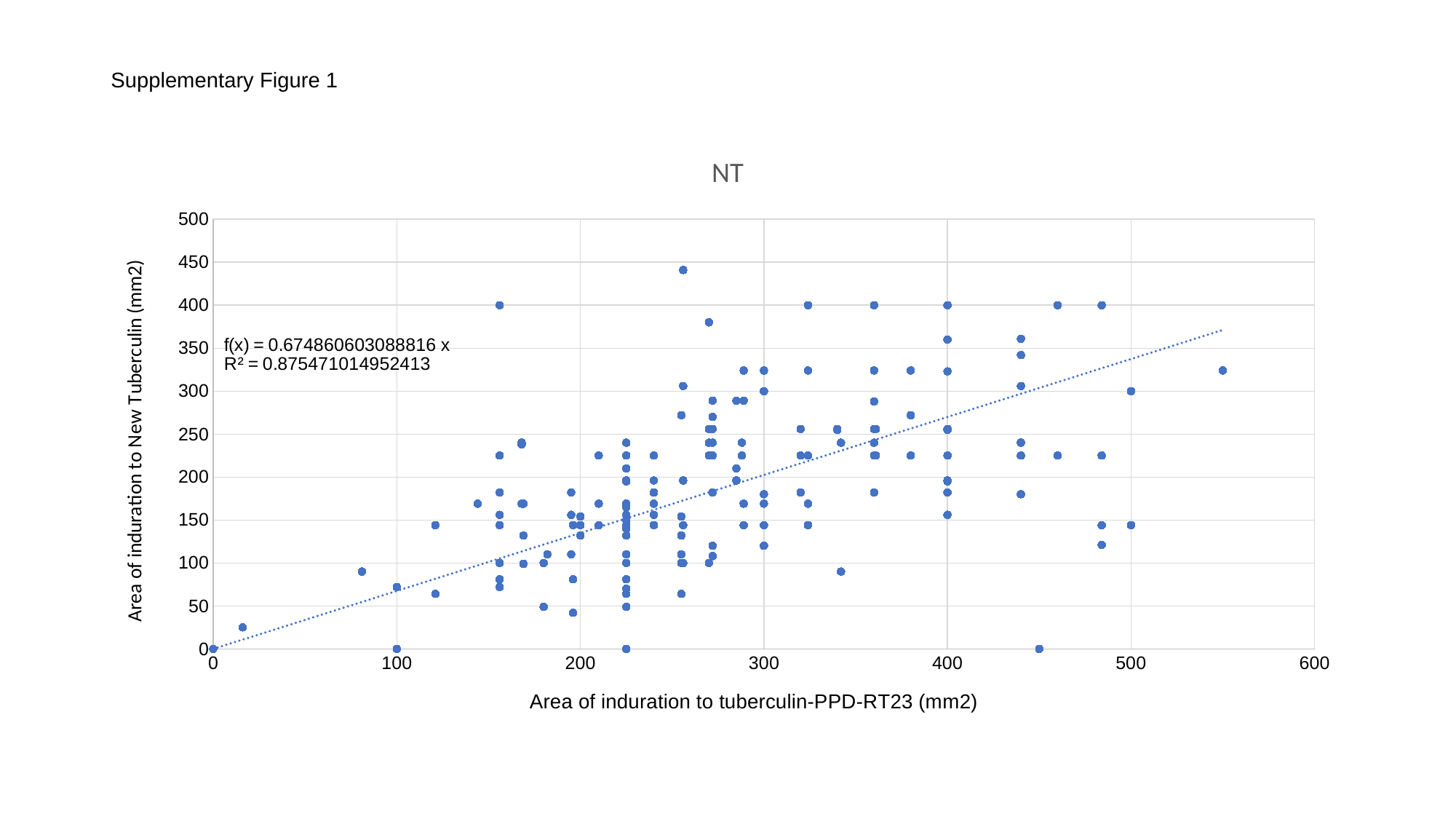

# Supplementary Figure 1
### Chart:
| Category | NT |
|---|---|

Supplement: Supplementary file 2 [file Presentation_1.PPTX]
